# Awareness, attitudes towards genetic diseases and acceptability of genetic interventions among pregnant women in Burera district, Rwanda

**DOI:** 10.1186/s12889-023-16866-3

**Published:** 2023-10-10

**Authors:** Jean Baptiste Niyibizi, Erigene Rutayisire, Monica Mochama, Michael Habtu, Zephanie Nzeyimana, Daniel Seifu

**Affiliations:** 1 School of Public Health, Mount Kenya University, Kigali Campus, Rwanda; 2 School of Medicine, Basic Medical Sciences Division, University of Global Health Equity (UGHE), Butaro, Rwanda; 3 School of Public Health, University of Rwanda, Kigali, Rwanda; 4 ICAP Global Health, Columbia University, Kigali, Rwanda

**Keywords:** Genetic diseases, Awareness, Attitude, Acceptability, Genetic interventions

## Abstract

Approximately 3% of all pregnancies are associated with conditions linked with disability, either mild or severe congenital diseases. This is a consequence of environmental and genetic exposures. Complications and poor management of these diseases arise due to limited knowledge, awareness about the disease, and limited resource settings. The current study assessed awareness, attitudes towards genetic diseases, and acceptability of genetic interventions among pregnant women. This was a cross-sectional study that was conducted among 664 pregnant women in six selected health centers in Burera district using a detailed questionnaire. The data were analysed using STATA Version 15 and entailed univariate, bivariate, and multivariable analyses. The level of significance was set at p < 0.05. The mean age of the study participants was 28, and most of them were in the age range of 21 to 30 (50%). Most of the participants were married (91.1%), Christians (98.4%), farmers (92.7%), used public health coverage (96.6%), and attained primary studies (66.1%). The findings from this study showed that among participants, adequate awareness was at 29.5%, inadequate awareness at 70.5%, positive attitudes at 87.1%, negative attitudes at 12.9%, high acceptability at 97.1%, and low acceptability at 2.9%. While there was no significant difference between awareness and acceptability, there was a statistical significance between attitudes towards genetic diseases and acceptability towards the use of genetic services (p < 0.01). There was no statistical significance between sociodemographic or obstetric characteristics and the acceptability of genetic interventions. Participants with positive attitudes towards genetic diseases were more likely to develop a high level of acceptability and willingness towards the use of genetic interventions (OR: 5.3 [2.1–13.5]). Improving awareness about genetic diseases and establishing genetic interventions in healthcare facilities are needed.

**Supplementary Information:**

The online version contains supplementary material available at 10.1186/s12889-023-16866-3.

## Introduction

Genetic diseases are distributed throughout the world, and there are 20.40 birth defects for every 1,000 babies born in sub-Saharan Africa [1]. A genetic disease or disorder is any disease caused in part or in whole by a mutation in the DNA sequence of an individual away from the normal DNA sequence [2] and [3]. Genetic disorders can be multifactorial (caused by multiple gene mutations), which can result from a combination of gene mutations and environmental variables or chromosomal damage (alterations in the number, structure, or shape of full, complete chromosomes or gene-carrying structures) [4]. In some cases, this occurs during meiosis. In other cases, it is present in the parent’s cells that are passed on to the offspring. Genetic diseases can be passed on through generations and inherited from parents through the germ line (inherited genetic diseases); other diseases can arise spontaneously during conception; and other genetic diseases involve genes that arise during the course of an individual’s lifetime (mostly cancer). If the disorder is found on the autosomes, it is referred to as an autosomal condition, and if it is found in sex chromosomes, the condition is called a sex-linked condition (x- or y-linked) [5].

Congenital genetic diseases are diseases that are born due to inheritance from parents or environmental exposures during conception, embryogenesis, or fetal development. The most well-known defect is a neural tube defect that is due to a deficiency of vitamin B9, known as folic acid [6]. Smoking during pregnancy can harm the unborn baby’s tissues, particularly the lungs and brain. Many studies have linked maternal smoking and cleft lip. Tobacco smoke contains carbon monoxide, which can prevent a growing infant from obtaining adequate oxygen. Furthermore, tobacco smoke can also cause miscarriage, and it contains other chemicals that can harm the baby. Moreover, smoking was found to be associated with congenital heart defects (CHDs) [7]. Alcohol can have serious effects on the fetus, such as neurological defects (cognitive impairment, learning disabilities, or behavioral abnormalities), growth retardation, and facial anomalies. The destruction of fetal and newborn red cells by maternal red cell antibodies specific for paternally inherited antigens on fetal red cells or erythroid precursors is characterized as Hemolytic Disease of the Fetus and Newborn (HDFN) [8]. HDFN affects approximately 500 babies in England each year and can result in stillbirths, disability, or death due to anemia and jaundice [9].

In the last more than 100 years, Down syndrome has been the most common genetic disorder that has led to mental retardation, specific birth defects, decreased life expectancy, and other disease conditions [10]. Down syndrome is most prevalent in the United States of America, where 1 in almost every 700 newborn babies has this disorder [10] and [11]. In Tunisia, a high prevalence of consanguinity was reported, and 346 different genetic disorders were identified [12]. In the study performed by Molteno et al., 1997, Down syndrome was found to be common, with a prevalence of 1.3% in South Africa [13]. According to 25 studies from nine sub-Saharan African countries in a systematic study performed by Adane et al., 2020, the southern African region has the highest rate of genetic birth disorders, with 43 birth problems per 1000. Birth abnormalities in the musculoskeletal system were reported to have a pooled prevalence of 3.90 per 1000 in the same study. Birth defects were highly associated with a lack of folic acid supplementation, the incidence of chronic disease, and drug use during pregnancy. In the study survey performed in Rwanda by Mutesa et al., 2010, the prevalence of different genetic diseases was identified among 345 patients. In this study, it was found that the most prevalent genetic disease was Trisomy 21 (18.26%). Other identified syndromes include Robertsonian translocation 21 (0.58%), Edward syndrome or trisomy 18 (0.87%), and Patau syndrome or trisomy 13 (1.16%). The least prevalent among the surveyed diseases studied were Trisomy 10p, deletion 13q, mosaic Turner syndrome, Cat eye syndrome, mosaic cat eye syndrome and balanced translocation, both of which were 0.29% [14].

The most common and routine reason to refer a pregnant woman for prenatal diagnosis is when an anomaly is identified during an ultrasound scan of the fetus. Another rare reason is a high-risk result from screening tests on maternal blood during pregnancy. It is worth noting that almost all mothers older than 39 are in danger or at risk of transmitting or transmitting a genetic disorder to their offspring, so women of this age should seek genetic testing during their pregnancy. Other common referrals for genetic testing include a family history of genetic disease. Discrimination against people with chronic disabilities (primarily caused by genetics) might take the form of job rejection, health insurance denial, or just social acceptability. This leads to the marginalization of these individuals and significantly compromises the principles and goals of public health. It is worth noting that integrated clinical and social support systems, which incorporate genetic disease and predisposition counselling for patients and their families, can lead to better patient care and treatment as well as an improved quality of life [15].

Genetic information or results usually have an impact not only on the person being tested but also on their relatives across generations. One of the numerous applications of genetic testing is to assist couples in becoming aware of the hereditary traits of their born and unborn children. Another benefit of genetic tests is that they allow people to learn about their hereditary propensity for disease [2]. In many African countries, including Rwanda, prenatal genetic testing and premarital genetic screening for couples are not in place. In most cases, prenatal diagnosis includes testing for some infectious diseases, such as HIV and syphilis, as well as for the ABO and Rh blood groups. However, screening for genetic diseases is not done, mainly because the facilities and required resources to carry out genetic testing are not available or adequate. It is obvious that if genetic services are not in place, genetic counselling could not be implemented as well. However, at least genetic history-taking and a little counselling based on the history should be done. In an endeavor to diminish hereditary diseases, many communities and other stakeholders encourage couples to undergo genetic testing before getting married and on the fetus during pregnancy to evaluate any disease risk [15, 16].

Awareness and attitudes vary internationally across continents, countries, and regions. In addition, these variables vary again according to different types of people, communities, and particular social determinants of health. In a study performed worldwide by Henneman et al., 2013, the results from study participants showed that the expectation of genetic and genome improvement achieves its goals with time [17]. The results from this study convinced genetic experts that genetic testing is being applied and that understanding genetic illnesses assists people in living longer lives; thus, testing should be applied widely and intensively. These positive results were made in 2002 and 2010, just after 10 years of the Human Genome Project implementation. In fact, in 2002 and 2010, 817 (63%) and 978 (70%) participants responded positively, respectively. The authors, however, highlight that worries about inequities remain with regard to insurance coverage. The study, again in 2010, found that people were more interested in knowing their genetic make-up.

While there are studies done on genetics and genetic diseases, there is no single research done in Rwanda about awareness, attitudes, and acceptability towards genetic diseases or genetic intervention. The lack of genetic awareness and positive attitudes towards genetic testing pose consequences for newborns, including congenital genetic diseases that would have been prevented, taken care of, or prepared psychologically using genetic counselling for parents, children, the entire family, and throughout generations at large. Moreover, the acceptability of genetic intervention is not yet known, and this could inform policy on the way forward in establishing and improving genetic services. Thus, the aim of this study was to assess awareness, attitudes towards genetic diseases, and acceptability of genetic interventions among pregnant women in Burera District, Rwanda.

## Research methodology

### Study design, setting, data collection, and variables

This study was a cross-sectional study design that was conducted on 664 pregnant women in six (6) selected health centers in Burera district, located in the northern province of Rwanda. The sample size was calculated using the Fisher et al. formula [18]. The study applied a quantitative approach in which a sampling method with multiple stages (multistage sampling technique) was used. In the first stage, six health centers were selected randomly, and then in the second stage, pregnant women visiting Antenatal Care (ANC) were selected systematically (every second pregnant woman was considered). However, the starting point was selected from the two (2) first pregnant women. The main tool of data collection was a questionnaire that was reviewed and validated by the research team (principal investigator and coprincipal investigators). It comprises five major sections: demographics questions, obstetric questions, awareness questions, attitudes questions, and acceptability of genetic interventions such as establishing and improving genetic services in the areas of screening, testing, and counselling. A Likert scale score of 1 to 3 was used to collect data on awareness. Attitudes ranged from strongly agree, agree, neutral, to strongly disagree. Acceptability was collected as either yes or no.

### Statistical analyses

Data were entered in a database created using MS Excel and then exported to STATA Version 15. Quantitative data analysis entailed descriptive/univariate, bivariate, and multivariate analyses. Descriptive analyses were computed using frequency, percentages, the mean, and the standard deviation to summarize the basic characteristics of the respondents. The awareness assessment focused on types of genetic disorders, causes, transmission, and prevention of genetic diseases. For the awareness assessment score, after aggregating, those who scored 77.77% and above were categorized as having adequate awareness, and those below 77.77% were categorized as having inadequate awareness. For attitudes, the respondents who scored 71.42% and above were classified as having positive attitudes, whereas those who scored below 71.42% were considered to have negative attitudes. For acceptability level towards use of genetic interventions, respondents who scored 71.42% and above (5 and above out of 7) were classified as having a high acceptability level towards use of genetic interventions, whereas those who scored below 71.42% were considered to have a low acceptability level towards use of genetic interventions. In bivariate analysis, a chi-square test was applied to assess the association between dependent and independent variables. Multivariable analysis was performed to control confounding variables and determine the strength of the association. The significance level was set at 5%. The data are presented using tables.

## Results

### Demographic and obstetric characteristics of the study participants

The demographic and obstetric characteristics of the study participants are described in Table 1.

**Table 1:** Demographic and obstetric characteristics of the study participants

Characteristics	Outcome n (%)	Taking alcohol	Outcome n (%)
**Age, Mean (Min, Max)**	**28 (16–48)**	Yes	103(15.5)
Age groups		No	561(84.5)
16–20	94(14.2)	**My husband takes alcohol**	
21–30	332(50.0)	Yes	269(40.5)
31–40	196 (29.5)	No	395(59.5)
41–49	42(6.3)	**Frequently eat vegetables**	
**Education**		Never or rarely	486(73.2)
No formal education	130 (19.6)	More often	178(26.8)
Primary	438(66.1)	**Frequently eat fruits**	
Secondary and above	95(14.3)	Never or rarely	443(66.7)
**Marital status**		More often	221(33.3)
Single	54(8.1)	**Receiving iron supplement from Health Facility**	
Married	605(91.1)	Yes	664(100)
Divorced/separated	5(0.8)	No	0
**Religion**		**Reception of Vit B9 supplement**	
Christian	653(98.4)	Yes	0
Muslim	11(1.7)	No	664(100)
**Economic category**		**Having a small vegetable yard at home**	
I	117(17.7)	Yes	544(81.9)
II	369(55.6)	No	120(18.1)
III	178(26.9)	**Obstetric characteristics**	**Outcome n (%)**
**Occupation**		Have you ever had any birth defects	
Farmer	615(92.7)	Yes	11(1.7)
Civil servant	20(3.0)	No	653(93.4)
Private and Business	29(4.4)	Have you ever had Still birth	
**Health insurance**		Yes	17(2.6)
Public health cover	641(96.6)	No	647(97.5)
Civil servant and private health cover	23(3.5)	Pregnancy trimester	
Ever diagnosed a GD		I	198(29.9)
Yes	5(0.7)	II	208(31.4)
No	659(99.3)	III	258(38.7)
**Any relative (in at least 3 generations) diagnosed with GD**		Number of pregnancies	
Yes	0–2	360(54.2)	
No	6-Mar	269(40.5)	
**Living with a chronic disease**	7+	35(5.3)	
Yes	4(0.6)	Number of parities	
No	660(99.4)	0–2	480(72.3)
**Smoking**		6-Mar	168(25.3)
Yes	1(0.2)	7+	16(2.5)
No	663(99.8)	First ANC trimester	
**My partner smoke**		I	386(58.2)
Yes	15(2.3)	II	170(25.6)
No	649(97.7)	III	98(14.8)

*GD: Genetic disease

Table 1 shows the demographic characteristics of the study participants. Most of the participants were in the age group of 21–30 (50%), had attained primary school (66.1%), were married (91.1%), were Christians (98.4%), were in social economic category II (55.6%), were farmers (92.7%), and many used public health coverage (96.6%). Very few participants were diagnosed with GD (0.7%), a few had relatives diagnosed with GD (7.3%), very few participants were smokers (0.2%), and a few were taking alcohol (15.5%). Although many people have a small yard of vegetables at home, a small number frequently take vegetables (26.8%) and fruits (33.3%). Health facilities regularly provide iron to pregnant women, but they have not yet started the provision of folate. The table also shows the obstetric characteristics of the study participants. Most of the participants did not have birth defects (93.4%), did not have stillbirths (97.5%) and were in the third trimester of gestation (38.7%). Many pregnant women reported having 3–6 pregnancies; however, the total delivery peak was between 0 and 2 (72.3%). This means that there were some significant abortions among the participants. In addition, many pregnant women who visited antenatal care services were in their first trimester (58.2%).

Rwanda’s socioeconomic state is measured using the poverty social category developed by the Minister of Local Government and Social Affairs in 2015. There are four income groups, with the first being extremely poor, the second poor, the third self-sustaining, and the fourth prosperous [19].

### Awareness of the study participants

The awareness of the study participants is described in Table 2.

**Table 2:** Awareness of the study participants

Characteristics	n (%)
**General awareness about genetic diseases**	
**Ever heard about genetic disease**	
Yes	332(50)
No	332(50)
**Whether know any genetic disease**	
Yes	307(46.2)
No	357(53.8)
**Are genetic diseases contagious/infectious**	
Yes	159(23.9)
No	505(76.1)
**Whether aware of existence of genetic tests**	
Yes	312(46.9)
No	352(53.1)
**Knowledge of risks/exposures towards developing GD for a pregnant woman**	
**Marriage between closed relatives**	
Yes	453(68.2)
No	211(31.8)
**Smoking for pregnant woman**	
Yes	383(57.7)
No	281(42.3)
**Exposure of secondary smoke to pregnant woman**	
Yes	324(48.8)
No	340(51.20)
**Taking alcohol during pregnancy**	
Yes	293(44.1)
No	371(55.9)
**Not eating a balanced diet for a pregnant woman?**	
Yes	581(87.5)
No	83(12.5)

*GD: Genetic disease

Table 2 demonstrates the awareness level score among the study participants. The true answer was scored 1, whereas the wrong answer was scored zero. The overall awareness score was out of 9. Scores less than 7 were considered inadequate awareness, whereas scores of 7 and above were considered adequate awareness. The results showed that the overall proportion of respondents who had an adequate awareness score was 29.5%, whereas 70.5% of the participants had inadequate awareness. The results also showed that half of the participants heard about GD. More than half (53.8%) of the participants did not know of any example of a genetic disease. A good percentage of participants (23.9%) thought that genetic diseases were contagious. A total of 53.1% of the participants were not aware of the genetic tests that could be applied in genetic testing. Although more than half of the participants have good knowledge about risks that could lead to GD for a pregnant woman, a great number of participants believe that taking alcohol (55.9%) and exposure to secondary smoke (51.2%) cannot cause a risk of developing a genetic defect in a pregnant woman.

### Attitudes of the study participants

The attitudes of the study participants are described in Table 3.

**Table 3:** Attitudes of the Study Participants

Variables	n (%)
I support prenatal genetic testing as a way of genetic disease screening	
Yes	644 (97.0)
No	20 (3.0)
I can consent for abortion in case a serious genetic disease is found in my fetus	
Yes	147 (22.1)
No	517 (77.9)
I think it is good to screen asymptomatic children for genetic diseases they are at high risk of	
Yes	544 (81.9)
No	120 (18.1)
I think it is necessary to go for genetic testing for a couple before getting married	
Yes	644 (97.0)
No	20 (3.0)
I think couples should stop marriage in case of any serious genetic disease identified	
Yes	491 (73.9)
No	173 (26.1)
It is good to screen a population for genetic diseases	
Yes	645 (97.1)
No	19 (2.9)
I believe genetic counselling is vital	
Yes	658 (99.0)
No	6 (1.0)

Table 3 describes the attitudes of the study participants. A positive attitude was scored 1, whereas a negative attitude was scored zero. The overall awareness score was out of 7. Scores less than 5 were considered negative attitudes, whereas scores of 5 and above were considered positive attitudes. The overall positive attitude score among study participants was 87.1%, whereas the overall negative attitude score was 12.9%. In addition, it was found that many pregnant women support prenatal genetic testing (97%) as a method of genetic disease screening for the fetus, even if the anomaly is not found during ultrasound. Many participants (22.1%) in this study revealed that they cannot consent to abortion in case a fetus is found to have a serious disease that may cause death in utero or early in life. Many pregnant women felt that it was important to screen asymptomatic children for genetic diseases they were at risk of, even if there was no current treatment or prevention in place. Surprisingly, a very few participants (3%) agree that genetic screening for couples is necessary before getting married, and only 26.1% of couples should stop marriage if a genetic disease is found while screening. Moreover, only 1% of the participants believe genetic counselling is important.

### Acceptability or willingness of genetic interventions

The acceptability or willingness of genetic interventions is described in Table 4.

**Table 4:** Acceptability or willingness of genetic interventions

Variables	Outcome n (%)
I would wish to use genetic testing if available	
Yes	657 (98.9)
No	7 (1.1)
I would move to another health facility where genetic services can be accessed	
Yes	647 (97.4)
No	17 (2.6)
I would be happy if genetic services are integrated in routine healthcare services	
Yes	652 (98.2)
No	12 (1.8)
If a genetic disease is identified during my pregnancy, I would consent for preimplantation diagnosis and In vitro fertilization for the next pregnancy	
Yes	251 (37.8)
No	413 (62.2)
I can consent for my child to be tested/screened for genetic disorders	
Yes	653 (98.3)
No	11 (1.7)
I would wish to use genetic counselling services if available	
Yes	656 (98.8)
No	8 (1.2)
If natural conception means fail, I can consent for preimplantation using invitro fertilization	
Yes	319 (48.0)
No	345 (52.0)

Table 4 describes the acceptability of genetic interventions among respondents. The true answer was scored 1, whereas the wrong answer was scored zero. The overall acceptability score was out of 7. Scores less than 5 were considered low acceptability, whereas scores of 5 and above were considered high acceptability. According to the results found, there was a high acceptability level among participants (97.1%), whereas only a low percentage (2.9%) showed a low level of awareness. In addition, according to the table, 97.4% of the participants confirmed that if needed, they would transfer to another health facility that provides genetic services. A good number (98.8%) of the participants would use genetic counselling if available at a health facility. In addition, 48% of the participants would use preimplantation using in vitro fertilization for subsequent pregnancies if the first one was identified to have a genetic disease that would probably be heritable or at risk of being passed on through generations. Furthermore, regarding failure to conceive naturally, 62.2% of the pregnant women consented that they should go for any means of in vitro fertilization that would be in place and in accordance with the possible problems that could be identified during diagnosis, including genetic disease.

### Awareness, attitudes, and acceptability categories by participants’ characteristics

Table 5 demonstrates the awareness, attitudes, and acceptability categories based on the participants’ characteristics.

**Table 5:** Awareness, attitudes, and acceptability categories by participants’ characteristics

Variables	*Awareness*				*Attitudes*				*Acceptability*			
	Inadequately aware, n(%)	Adequately aware, n (%)	N	p Value	Negative attitudes, n(%)	Positive attitudes, n(%)	N	p Value	Low acceptability, n(%)	High acceptability, n (%)	N	p Value
**Demographic characteristics**												
Age groups												
16–20	65(69.2)	29(30.8)	94	0.953	4(4.2)	90(95.8)	94	0.019	2(2.1)	92(97.9)	94	0.539
21–30	233(70.2)	99(29.8)	332		47(14.2)	285(85.8)	332		12(3.6)	320(96.4)	332	
31–40	139 (70.9)	57(29.1)	196		32(16.3)	164(83.7)	196		5(2.5)	191(97.5)	196	
41–49	31 (73.8)	11(26.2)	42		3(7.2)	39(92.8)	42		0	42(100)	42	
Education												
No formal education	116(89.2)	14(10.8)	130	< 0.01	25(19.2)	105(80.8)	130	0.01	4(3.1)	126(96.9)	130	0.518
Primary	295(67.3)	143(32.7)	438		44(10.1)	394(89.9)	438		14(3.2)	424(96.8)	438	
Secondary and above	56(58.9)	56(41.1)	95		16(16.8)	79(83.2)	95		1(1.1)	94(98.9)	95	
Religion												
Christian	458(70.2)	195(29.8)	653	0.134	82(12.5)	571(87.5)	653	0.02	19(2.9)	634(97.1)	653	0.566
Muslim	10(90.9)	1(9.1)	11		4(36.4)	7(63.6)	11		0	11(100)	11	
Occupation												
Farmer	429(69.8)	186(30.2)	615	0.165	72(11.7)	543(88.3)	615	< 0.01	16(2.6)	599(97.4)	615	0.337
Civil servant	14(70)	6(30)	20		8(40)	12(60)	20		1(5.0)	19(95.0)	20	
Private and Business	25(86.2)	4(13.8)	29		6(20.7)	23(79.3)	29		2(6.9)	27(93.1)	29	
Health insurance												
Public health cover	453(70.7)	188(29.3)	641	0.573	78(12.2)	563(87.8)	641	< 0.01	19(2.9)	622(97.1)	641	0.402
Civil servant and private health cover	15(65.2)	8(34.8)	23		8(34.8)	15(65.2)	23		0	23(100)	23	
**Obstetric characteristics**												
Ever had birth defects												
Yes	9(81.8)	2(18.2)	11	0.406	4(36.3)	7(63.7)	11	0.02	1(9.1)	10(90.9)	11	0.211
No	459(70.3)	194(29.7)	653		82(12.5)	571(87.5)	653		18(2.8)	635(97.2)	653	
Ever had Still birth												
Yes	14(82.3)	3(17.7)	17	0.277	7(41.2)	10(58.8)	17	< 0.01	0	17(100)	17	0.473
No	454(70.2)	193(29.8)	647		79(12.2)	568(87.8)	647		19(2.9)	628(97.1)	647	
Current pregnancy trimester												
I	112(56.5)	86(43.5)	198	< 0.01	9(4.5)	189(95.5)	198	< 0.01	4(2.1)	194(97.9)	198	0.735
II	157(75.5)	51(24.5)	208		16(7.7)	192(92.3)	208		8(3.8)	200(96.2)	208	
III	198(77.0)	59(23)	257		61(23.7)	196(76.3)	257		7(2.7)	250(97.3)	257	
First ANC trimester												
I	262(67.9)	124(32.1)	386	< 0.01	70(18.2)	316(81.8)	386	< 0.01	13(3.4)	373(96.6)	386	0.604
II	128(75.3)	42(24.7)	170		14(8.2)	156(91.8)	170		5(2.9)	165(97.1)	170	
III	75(76.5)	23(23.5)	98		2(2.1)	96(97.9)	98		1(1.0)	97(99.0)	98	
Any relative (in at least last 3 generations) diagnosed with GD												
Yes	23(47.9)	25(52.1)	48	< 0.01	3(6.2)	45(93.8)	48	0.151	1(2.1)	47(97.9)	48	0.737
No	445(72.3)	171(27.8)	616		83(13.5)	533(86.5)	616		18(2.9)	598(97.1)	616	
Smoking												
Yes	1(100)	0	1	0.517	1(100)	0	1	< 0.01	0	1(100)	1	0.864
No	467(70.5)	196(29.5)	663		85(12.8)	578(87.2)	663		19(2.8)	644(97.2)	663	
My partner smoke												
Yes	13(86.7)	2(13.3)	15	0.165	7(46.7)	8(53.3)	15	< 0.01	1(6.6)	14(93.4)	15	0.371
No	455(70.1)	194(29.9)	649		79(12.2)	570(87.8)	649		18(2.7)	631(97.3)	649	
Frequently eat vegetables												
Never or rarely	345(70.9)	141(29.1)	486	0.637	76(15.6)	410(84.4)	486	< 0.01	15(3.1)	471(96.9)	486	0.566
More often	123(69.1)	55(30.9)	178		10(5.6)	168(94.4)	178		4(2.2)	174(97.8)	178	
Frequently eat fruits												
Never or rarely	321(72.5)	122(27.5)	443	0.114	76(17.2)	367(82.8)	443	< 0.01	14(3.2)	429(96.8)	443	0.513
More often	147(33.5)	74(33.5)	221		10(4.5)	211(95.5)	221		5(2.2)	216(97.8)	221	
Having a small vegetable yard at home												
Yes	371(68.2)	173(31.8)	544	< 0.01	70(12.8)	474(87.2)	544	0.891	17(3.2)	527(96.8)	544	0.386
No	97(80.8)	23(19.2)	120		16(13.3)	104(86.7)	120		2(1.7)	118(98.3)	120	
TOTAL	468(70.5)	196(29.5)	664		86(12.9)	578(87.1)	664		19(2.9)	645(97.1)	664	

Table 5 shows that awareness of GD is influenced by the level of education, history of GD in the family and possession of small yards at home (p < 0.05, specifically all have p < 0.01). Regarding attitudes, it was found that attitudes were strongly affected by age, religion, occupation, health insurance, history of birth defects, stillbirth, and gestational trimester (p < 0.05). In terms of acceptability or willingness to use genetic services, it was found that the level of acceptability or willingness was not affected by participant characteristics (neither demographic nor obstetrics), p > 0.05.

### Logistic regression of factors associated with awareness, attitudes, and acceptability towards GD and genetic interventions

Table 6 describes the logistic regression of factors associated with awareness, attitudes, and acceptability.

**Table 6:** Logistic regression of factors associated with awareness, attitudes, and acceptability towards genetic diseases and genetic interventions

A. Awareness				
Variables		95% CI		P value
	aOR*	Lower	Upper	
**Education**				
No formal education	1			
Primary	3.8	2.0	6.8	˂0.01
Secondary and above	5.9	2.9	12.0	˂0.01
First ANC trimester				
I	1			
II	0.7	0.5	1.1	0.104
III	0.5	0.3	0.9	0.042
Any relative (in at least last 3 generations) diagnosed with GD				
Yes	1			
No	0.3	0.2	0.6	˂0.01
**B. Attitudes**				
**Age groups**				
16–20	**1**			
21–30	0.3	0.1	0.8	0.018
31–40	0.3	0.1	0.9	0.028
41–49	0.8	0.2	3.7	0.749
**Education**				
No formal education	1			
Primary	2.2	1.2	4.0	0.006
Secondary and above	1.2	0.6	2.5	0.636
**Religion**				
Christian	1			
Muslim	0.2	0.1	0.8	0.019
Have you ever had any birth defects				
Yes	1			
No	0.3	0.1	1.2	0.088
Have you ever had Still birth	1			
Yes	1			
No	0.2	0.1	0.6	0.004
My partner smoke				
Yes	1			
No	4.7	1.6	14.2	0.006
Frequently eat vegetables				
Yes	3.1	1.5	6.3	0.002
No	1			
**C. Acceptability/willingness**				
Marriage between closed relatives				
Yes	1.9	0.7	5.0	0.202
No	1			
Exposure of secondary smoke to pregnant woman				
Yes	2.2	0.7	6.5	0.16
No	1			

As illustrated in Table 6, it was found that the odds of developing adequate awareness for participants with primary education were 3.8 times higher than those with no formal education. Subsequently, the odds of developing adequate awareness for participants with secondary education were 5.9 times higher than those with no formal education. Moreover, the odds of developing adequate awareness for participants with no history of genetic diseases were 0.3 less likely than those with a history of genetic diseases (aOR: 0.3, CI: 0.2–0.6, and p < 0.01). The results also showed that participants aged 16–20 were more likely to develop adequate attitudes compared to the rest of the age groups. It was also noted that the odds of developing positive attitudes in pregnant women with primary education were 2.2 times more likely than those with no formal education (aOR: 2.2, CI: 1.2-4).

Again, the odds of developing positive attitudes in Muslims were 0.2 times less likely than in Christians (aOR: 0.2, CI: 0.1–0.8) compared to their fellow Christians. From the same table, the odds of developing positive attitudes in pregnant women who never experienced stillbirth were 0.2 times less likely than those who experienced it (aOR: 0.2, CI: 0.1–0.6). The odds of developing a high acceptability level in pregnant women who supported marriage between close relatives were 1.9 times more likely than those who discouraged marriage between close relatives.

### Association between awareness and attitudes in relation to the acceptability of genetic interventions

Table 7 compares the influence of awareness and attitudes towards the acceptability of genetic testing and counselling services among participants.

**Table 7:** Association between awareness and attitudes in relation to the acceptability of genetic interventions

Variables	Low acceptability, n (%)	High acceptability, n (%)	N	p Value
Awareness towards genetic diseases				
Inadequate (Score of less than 68%)	17(3.6)	451(96.4)	468	0.066
Adequate (Score of 68% and above)	2(1.0)	194(99.0)	196	
Attitudes towards genetic diseases				
Negative (Score of less than 68%)	8(9.3)	78(90.7)	86	˂0.01
Positive (Score of 68% and above)	11(1.9)	567(98.1)	578	
Total	19(2.9)	645(97.1)	664	

Table 7 shows an association between awareness and attitudes in relation to the acceptability of genetic interventions, and it was found that there was a statistical significance between attitudes towards genetic diseases and acceptability towards genetic services (p < 0.01). The awareness level was not associated with the acceptability or willingness level towards the use of genetic services (p > 0.05).

### Association between awareness and attitudes in relation to the acceptability of genetic interventions

**Table 8:** Logistic regression between attitudes and acceptability of genetic services

Variables	Adjusted Odds Ratio	95% CI		P value
		Lower	Upper	
**Awareness (Overall score)**				
Adequate (score of 7 and above)	3.45	0.9	6.7	0.076
Inadequate (Score less than 7)	1.00			
**Attitudes towards genetic diseases**				
Positive (Score of 5 and above)	5.30	2.1	13.5	˂0.001
Negative (Score less than 5)	1.00			

*Predicted probabilities are of membership of high acceptability of genetic services

The findings from Table 8 demonstrated that the odds of developing a high acceptability level towards the use of genetic services in pregnant women with positive attitudes were 5.3 times more likely than those with negative attitudes (aOR: 5.3 [2.1–13.5]).

## Discussion

In the current study, most of the participants were in the age group of 21–30 (Table 1). In addition, the awareness level of genetic disease was inadequate (at 70.5%), whereas only 29.5% proved to have adequate awareness (Table 2). Furthermore, the overall positive attitude score among research participants was 87.1%, and many pregnant women (97%) accepted prenatal genetic testing as a method of screening for genetic diseases in the fetus. However, only a small percentage of individuals (3%) believe that genetic screening for couples before marriage is important (Table 3). Moreover, the level of acceptability towards the utilization of genetic services was 97.1%. In addition, 98.9% of the pregnant women agreed that they would use genetic testing services if available, and 97.4% confirmed that they would consent to transfer to another health facility where the genetic services are offered (Table 4). There was an association between views and acceptance of genetic services but not between awareness and acceptance (Tables 7 and 8).

In a study conducted in Nigeria in 2018, it was found that 69.4% of the participants enrolled in the study had knowledge of genetics and genetic diseases. In the same enrolled participants, 97.6% had very little knowledge about prenatal genetic disease testing or screening, 23.9% had positive attitudes towards genetic testing, and only 10.1% supported termination (abortion) of the affected pregnancies [20]. These findings were different from those of the current study, where the awareness level was found to be 29.5% (Table 5). Only 22.1% of the participants in the current study consented to go for abortion in case a very serious genetic disease was identified in the foetus during pregnancy. In a study conducted in Saudi Arabia by Arafah et al., 2021, 62.7% of the participants responded that they could abort if the prenatal test showed that the embryo had a genetic disorder. It is evident that the decision on abortion could be affected by demographic and cultural characteristics. Furthermore, in this research performed by Arafah et al., 2021, married subjects (30%) were more willing to take part in the research than single (17%) subjects, which agrees with the current study, where 91.1% of the participants were married, whereas 8.1% were single (Table 1).

In the cross-sectional study conducted on the Visegrad countries’ understanding, awareness, and attitudes towards genetic testing, these countries showed slightly different attitudes and awareness towards the testing of genetic illnesses. The results of this study revealed that Hungarian citizens had a more positive view of the personal gains or benefits (with a mean of 3.64) of going to genetic testing, whereas Czech, Slovak, and Polish citizens followed with a mean of 3.36. It was found that Slovak citizens had quite a significant belief (mean of 2.78) compared to Hungarians (mean of 2.68). The Hungarian public demonstrated a high level of acceptance in regard to the availability and use of genetic testing (mean of 3.87), and the Czech people came in second among the four countries. Slovaks were the least supportive, with a mean of 3.36 [21]. Some of these agreed with the current study, where 97.1% of the participants showed that it is beneficial for a general population to undergo genetic testing (Table 3). In addition, the results from the current study revealed a high acceptability level (97.1%) towards the use of genetic services that include genetic testing and genetic counselling (Table 4).

The study conducted in Pakistan by Tariq et al., 2018, on the assessment of knowledge, attitudes, and practices among pregnant women in Pakistan highlighted the need for education about screening for congenital conditions [22]. This study revealed that pregnant women were not aware of congenital hypothyroidism (CH) and its implications for the lives of their children. In addition, they were not aware that an early diagnosis could help in treating the condition. Although the specific objectives were slightly different, these results agree with the current study, where many pregnant women showed inadequate awareness (70.5%) about genetic diseases. Some examples from the current study showed that half of the participants did not hear about genetic diseases, whereas only 46.2% knew some examples of genetic diseases. Again, some participants (23.9%) indicated that genetic diseases are contagious, which is totally wrong. A good number of pregnant women (53.1%) did not know that there are some laboratory tests that could be used to diagnose genetic diseases (Table 2).

Attitudes and knowledge on genetic testing among Jordanians, conducted by Khdair et al., 2021, found that 65.4% of the participants had good knowledge about genetics [23]. The major predictors of this factual knowledge in this study were level of education, employment or study in a health-care context, and monthly earnings. However, the participants showed that they perceived low knowledge about medical uses, with 39.5% and 23.9% showing that genetic findings may have possible social implications or effects. Regarding attitudes, 91.5% of the participants showed positive attitudes towards genetic testing. The predictors of these positive attitudes were high education and participants with better factual knowledge. Although the current study showed inadequate knowledge of genetic disease, it was found that the level of education, pregnancy trimester, first visit to ANC, having a family history of GD, and having a small yard of vegetables at home influenced the level of awareness and knowledge of genetic diseases (Table 5). Moreover, the level of education and first visit to the ANC were found to have a unique contribution to developing awareness about genetic diseases. The results regarding attitudes agreed with the current study, where 87.1% showed positive attitudes towards genetic diseases (Table 5). The major factors that influenced attitudes were age group, education, religion, history of birth defects, history of stillbirth, having a smoking partner, and frequency of vegetable intake. In particular, the age range, education level, religion, history of stillbirth, and frequency of vegetable intake had unique contributions to attitudes, as evidenced by the adjusted odds ratios and p < 0.05 shown in Table 6. It is worth noting that religion influence could have also been due to the overall number of Christians (98.4%) [Table 1].

In the study conducted by Ogamba et al., 2018, on genetic diseases and prenatal genetic testing, focusing on knowledge and attitudes in Nigeria, it was found that 69.4% of the participants did not have good knowledge about genetic diseases, whereas 61.8% were comfortable undergoing genetic testing. According to the findings, 23.9% of the participants had positive, better, or favorable attitudes towards genetic testing. Moreover, only 10.1% of the respondents supported the termination of affected pregnancies [20]. This study agrees with the current level of knowledge, whereas in the current study, the lack of knowledge about genetic diseases was high (Table 5). However, the current study demonstrated a good positive attitude (87.1%), and 98.9% of the pregnant women showed comfortability to undergo genetic testing if facilities and resources were allowed (Table 4). The current study showed that 22.1% could terminate pregnancy (almost more than half compared to the Ogamba et al., 2018 study).

The InterGEN study looked at the attitudes of African American moms toward genetic testing. A meta-data study showed that there is a major negative attitude toward genome studies, especially genetic testing of African origin. The results highlighted that from 2005 to 2015, all 80% of the participants were of European ancestry, whereas approximately 2.4% of the participants were of African origin. The same negative belief or attitude towards genetic testing was also observed in Americans of African ancestry. The study results also revealed that positive attitudes towards genetic testing were correlated with demographic factors such as higher levels of income. On the other hand, negative predictors of negative attitudes towards genetic testing were maternal age, country of origin, education, and religion [24]. Although in the current study, the level of negative attitudes was lower than that of positive attitudes, the results revealed that participants who attained primary education were found to have more positive attitudes. On the other hand, some religions (Muslim), pregnant women who never experienced stillbirth, participants aged between 21 and 30 and 31–40 and smoking history were found to be predictors of developing negative attitudes towards genetic diseases (Table 6). In another study conducted by Farsi et al., 2014, in an Oman region, 84.5% of the participants suggested that premarital carrier screening (PMCs) is necessary, whereas 49.5% supported that PMCs should be mandatory for everyone [25]. In another study conducted by Otovwe et al., 2019, in Nigeria, it was found that 50.6% of the participants had poor knowledge of marital genotype screening, while 63.20% had poor attitudes [26]. There was an established relationship between a family history of sickle cell disease (a genetic disease that runs in families) and the knowledge of the participants, who showed poor knowledge and negative attitudes towards genetic screening prior to getting married.

In the study done by Kvaratskhelia et al., 2021, on public attitudes toward genetic testing in Georgia, the majority of respondents showed that they are more curious about predictive genetic testing (75.3%); 40.6% of participants said they would rather get tested only for illnesses that can be treated or prevented [27]. In addition, in the same study, 65% of parents preferred that their children be screened for late-onset diseases, whereas 73% preferred carrier testing before conception. Furthermore, 59% of the respondents were not worried about the stigmatization of the disability results from genetic testing. These results agreed with the current study, where a great number (98.3%) could consent for their children to be screened for genetic diseases, whereas 97% of the participants confirmed that couples should undergo premarital genetic testing before marriage. In the current study, 86.7% of the pregnant women confirmed that genetic diseases in families could cause persistent familial conflicts, family plans and goals not being achieved, and breaking marriages (Tables 3 and 4). In another study conducted in Saudi Arabia by Arafah et al., 2021, the results showed that 87% of participants preferred getting tested before marriage, and the same number would not conceive if their child were predicted to fully suffer from genetic disorders [28].

In the study done in the Netherlands by Morren et al., 2007, on the perception of patients with chronic diseases’ genetic knowledge, attitudes toward genetic testing, and the link between these variables, they found that the genetic information was much lower in older people and less educated people [29]. These findings agree with those of the current study, where the odds of developing adequate awareness were 3.8 times higher for pregnant women who attained primary education than for those with no formal education (Table 6). In the same current study, the odds of developing adequate awareness were 5.9 times higher for pregnant women who had attained secondary education and above compared to those with no formal education (Table 6). Moreover, in the current study, it was statistically found that attitude level was statistically associated with the acceptability of genetic interventions (Tables 7 and 8).

In the study conducted by Maftei and Dănilă at Romania University in 2022 on attitudes towards genetic testing and implications for disability, there was variation among participants towards attitudes towards genetic testing [30]. In this study, religious individuals showed more concern for genetic testing as far as demographics were concerned. Furthermore, it was shown that regarding termination of pregnancy, information about having a deaf child comes first (35.8%) and major depressive disorder (MDD) (35.2% of respondents). Participants also showed different degrees of worry in relation to prenatal screening and ethical controversies. In the current study, the odds of developing a positive attitude towards genetic diseases were 0.2 times less likely compared to Christians (Table 6). Religion was not associated with developing adequate or inadequate awareness (Table 5). In addition, in the current study, there was no relationship between being religious and the development of high or low acceptability towards the use of genetic testing or counselling (Table 5). The findings by Maftei and Dănilă disagree to some extent with the current study regarding pregnancy termination and prenatal testing. In the current study, a great number (975%) of participants supported prenatal genetic testing as a method of genetic disease screening. However, a moderate percentage (22.1% of respondents) would consent to pregnancy termination if the fetus is at risk of serious genetic conditions (Table 3).

In a study performed by Adane et al., 2020, it was found that in sub-Saharan Africa, the prevalence of neural tube defects is 2.98 per 1000 newborns [1]. Neural tube defects and hydrops fetalis are rare disorders found in the fetus, and some of them are thought to be caused by underlying genetic conditions [31]. It was found that the genetic cause of some of these forms, including spina bifida or nonimmune hydrops fetalis, is more common in teenage mothers. In a study performed by Adane et al., 2020, it was found that neural tube defects are due to a lack of folic acid supplementation during pregnancy [1]. Although the current study did not focus on determining the prevalence of neural tube defects, it is worth noting that more participants were aged 21–30 (50%), and 14.2% were aged 16–20. The genetic cause of hydrops fetalis has been linked with a deficiency of folic acid (vit B9), which is essential in DNA synthesis. Teenage mothers frequently suffer from this deficiency, and subsequently, the fetus is at risk of developing a neural tube defect. In the current study, although all participants confirmed the receipt of iron (100%) from health facilities, 0% indicated that they did not receive folic acid (vitamin B9) from their health facilities. It is therefore recommended that the health facility and/or stakeholders in charge of nutrition advise on how the provision of vitamin B9 could be provided to young pregnant women to prevent this predisposition to developing hydrops fetalis. According to studies, pregnant women who include folic acid in their diets dramatically lower their risk of giving birth to a child with a neural tube defect. It is advised that all women of childbearing age take a daily vitamin supplement containing 400 micrograms of folic acid. Dark green vegetables, egg yolks, and select fruits are foods high in folic acid. A woman is more likely to become pregnant again with a neural tube defect if she already has a child with spina bifida, has spina bifida herself, or has had a pregnancy affected by any neural tube defect in the past. These women should take more folic acid as prescribed before and during the first trimester of pregnancy.

In the study conducted in Saudi Arabia by Arafah et al., 2021, 56.2% of the participants supported government running facilities for national citizens to be tested or screened for genetic diseases [28]. In the current study, 97.4% affirmed that they would accept transfers to go where genetic services are operational, and 98.2% supported that genetic services should be integrated into general health care services. This suggests a need to improve and establish genetic services that include carrier genetic screening, prenatal genetic screening, and genetic counselling in health care settings that are well channeled and accessed by many, especially those who are in need of using them.

## Conclusion

The study results revealed that the awareness level of genetic disease is 29.5%. Thus, education is needed for the public about the predisposition, inheritance, and management of genetic diseases. The level of acceptability and willingness towards the utilization of genetic testing and genetic counselling was 97.1%. This highlights that although participants have little awareness, they are willing to use genetic services. In addition, 98.2% of the participants wish to have genetic services integrated into general healthcare systems. Therefore, there is a need to improve genetic interventions in terms of screening, diagnosis, and management of genetic diseases to reduce and prevent associated morbidities and mortalities.

### Electronic supplementary material

Below is the link to the electronic supplementary material.

Supplementary Material 1

## Data Availability

All raw data and other processed data can be obtained from the corresponding author upon request.

## References

[1] Adane F, Afework M, Seyoum G, Gebrie A. Prevalence and associated factors of birth defects among newborns in sub-saharan african countries: a systematic review and meta-analysis. Pan Afr Med J. 2020;36. 10.11604/pamj.2020.36.19.19411 PMC7388615 32774596

[2] Strachan T. Human Molecular Genetics. Garland Sci. Mar. 2018;29:405–41. 4th ed.

[3] Watson JD (2014). Molecular Biology of the gene.

[4] Alberts B et al. *Molecular Biology of the Cell*. 6th ed., New York, Ny, Garland Science, 2015. pp. 943–972.

[5] Snustad DP, Simmons MJ (2016). Principles of Genetics.

[6] Greene NDE, Copp AJ. “Neural Tube Defects.” *Annual Review of Neuroscience*, vol. 37, no. 1, 8 July 2014, pp. 221–242, www.ncbi.nlm.nih.gov/pmc/articles/PMC4486472/, 10.1146/annurev-neuro-062012-170354 10.1146/annurev-neuro-062012-170354 PMC4486472 25032496

[7] Sullivan PM et al. “Risk of Congenital Heart Defects in the Offspring of Smoking Mothers: A Population-Based Study.” *The Journal of Pediatrics*, vol. 166, no. 4, Apr. 2015, pp. 978–984.e2, 10.1016/j.jpeds.2014.11.042. Accessed 9 Oct. 2020. 10.1016/j.jpeds.2014.11.042 25578997

[8] Harmening D (2019). Modern blood Banking & Transfusion Practices.

[9] National Institute for Health and Care Excellence (NICE). “Overview | High-Throughput Non-Invasive Prenatal Testing for Fetal RHD Genotype | Guidance | NICE.” *Www.nice.org.uk*, 9 Nov. 2016, www.nice.org.uk/guidance/dg25. Accessed 30 Dec. 2022.

[10] CDC. “Facts about down Syndrome.” *Centers for Disease Control and Prevention*, U.S. Department of Health & Human Services, 18 Nov. 2022, www.cdc.gov/ncbddd/birthdefects/downsyndrome.html. Accessed 5 Mar. 2022.

[11] SHerman SL et al. “Epidemiology of down Syndrome.” *Mental Retardation and Developmental Disabilities Research Reviews*, vol. 13, no. 3, 2007, pp. 221–227, genetics.emory.edu/documents/downsyndrome/sHerman_review_MRDDRR.pdf, 10.1002/mrdd.20157 10.1002/mrdd.20157 17910090

[12] Romdhane L, Abdelhak S. “Genetic Diseases in the Tunisian Population.” *American Journal of Medical Genetics Part A*, vol. 155, no. 1, 28 Dec. 2010, pp. 238–267, 10.1002/ajmg.a.33771. Accessed 8 Nov. 2021. 10.1002/ajmg.a.33771 21204241

[13] Molteno C et al. “Twenty-Year Birth Prevalence of down Syndrome in Cape Town, South Africa.” *Paediatric and Perinatal Epidemiology*, vol. 11, no. 4, Oct. 1997, pp. 428–435, 10.1046/j.1365-3016.1997.d01-25.x. Accessed 14 Mar. 2020. 10.1046/j.1365-3016.1997.d01-25.x 9373864

[14] Mutesa L et al. “A Survey of Genetic Diseases in Rwanda.” Rwanda Medical Journal, vol. 68, no. 3, Sept. 2010.

[15] WHO. “Genomics.” *Www.who.int*, 12 Nov. 2020, www.who.int/news-room/questions-and-answers/item/genomics. Accessed 12 Feb. 2021.

[16] Falahati AM et al. “Awareness and Attitude toward Genetic Counselling Services in South of Iran.” *Hormozgan Medical Journal*, vol. 23, no. 1, 31 Mar. 2019, p. e87158, www.semanticscholar.org/paper/Awareness-and-Attitude-Toward-Genetic-Counselling-Falahati-Nejatizadeh/ef042b8282c01b7e59db76af0c5c613fa22767e0, 10.5812/hmj.87158. Accessed 9 Sept. 2023.

[17] Henneman L et al. “Public Attitudes towards Genetic Testing Revisited: Comparing Opinions between 2002 and 2010.” *European Journal of Human Genetics*, vol. 21, no. 8, 1 Aug. 2013, pp. 793–799, www.ncbi.nlm.nih.gov/pmc/articles/PMC3722681/, 10.1038/ejhg.2012.271. Accessed 17 Aug. 2020. 10.1038/ejhg.2012.271 PMC3722681 23249955

[18] Althubaiti A. “Sample Size Determination: A Practical Guide for Health Researchers.” *Journal of General and Family Medicine*, vol. 24, no. 2, 14 Dec. 2022, 10.1002/jgf2.600 10.1002/jgf2.600 PMC10000262 36909790

[19] Williams T et al. “Distributing Social Transfers in Rwanda: The Case of the Vision 2020 Umurenge Programme (VUP).” *Research.manchester.ac.uk*, Oxford University Press, 9 June 2021, research. manchester.ac.uk/en/publications/distributing-social-transfers-in-rwanda-the-case-of-the-vision-20-2. Accessed 9 Sept. 2022.

[20] Ogamba CF et al. “Genetic Diseases and Prenatal Genetic Testing: Knowledge Gaps, Determinants of Uptake and Termination of Pregnancies among Antenatal Clinic Attendees in Lagos, Southwest Nigeria.” *Annals of Medical and Health Sciences Research*, vol. 8, no. 3, 2018, www.semanticscholar.org/paper/Genetic-Diseases-and-Prenatal-Genetic-Testing%3A-of-Ogamba-Roberts/ae6aa0b8dd5dd16280035b11bb8082aa99958e7d. Accessed 7 Oct. 2021.

[21] Bíró Klára et al. “Investigating the Knowledge of and Public Attitudes towards Genetic Testing within the Visegrad Countries: A Cross-Sectional Study.” *BMC Public Health*, vol. 20, no. 1, 10 Sept. 2020, 10.1186/s12889-020-09473-z. Accessed 6 May 2021. 10.1186/s12889-020-09473-z PMC7488256 32912246

[22] Tariq B et al. “Assessment of Knowledge, Attitudes and Practices towards Newborn Screening for Congenital Hypothyroidism before and after a Health Education Intervention in Pregnant Women in a Hospital Setting in Pakistan.” *International Health*, vol. 10, no. 2, 1 Mar. 2018, pp. 100–107, 10.1093/inthealth/ihx069. Accessed 15 June 2021. 10.1093/inthealth/ihx069 29528401

[23] Khdair SI et al. “Knowledge and Attitudes Regarding Genetic Testing among Jordanians: An Approach towards Genomic Medicine.” *Saudi Journal of Biological Sciences*, vol. 28, no. 7, July 2021, pp. 3989–3999, 10.1016/j.sjbs.2021.04.004. Accessed 4 June 2022. 10.1016/j.sjbs.2021.04.004 PMC8241592 34220256

[24] Wright ML et al. “African American Mothers’ Attitudes towards Genetic Testing in the InterGEN Study.” *Journal of Community Genetics*, vol. 11, no. 3, 7 Dec. 2019, pp. 285–290, 10.1007/s12687-019-00440-9. Accessed 2 Nov. 2021. 10.1007/s12687-019-00440-9 PMC7295918 31811592

[25] Al-Farsi, Omar A et al. “A Study on Knowledge, Attitude, and Practice towards Premarital Carrier Screening among Adults Attending Primary Healthcare Centers in a Region in Oman.” *BMC Public Health*, vol. 14, no. 1, 17 Apr. 2014, 10.1186/1471-2458-14-380. Accessed 22 May 2020. 10.1186/1471-2458-14-380 PMC4004421 24742222

[26] Otovwe A et al. “Knowledge and Attitude of Premarital Genotype Screening among Women of Child-Bearing Age in Kumo-Akko Local Government Area of Gombe State Nigeria.” *Semantic Scholar*, 13 Dec. 2019, www.semanticscholar.org/paper/Knowledge-and-Attitude-of-Premarital-Genotype-Among-Otovwe-Sunday/dc7ea204027052fc116e5be466426caeb49408e0. Accessed 2 Nov. 2021.

[27] Kvaratskhelia E et al. “Public Attitudes towards the Genetic Testing in Georgia.” *Journal of Community Genetics*, vol. 12, no. 3, 30 Mar. 2021, 10.1007/s12687-021-00522-7. Accessed 3 May 2021. 10.1007/s12687-021-00522-7 PMC8241973 33783754

[28] Arafah A et al. “Attitude and Awareness of Public towards Genetic Testing in Riyadh, Saudi Arabia.” *Saudi Journal of Biological Sciences*, vol. 28, no. 1, Oct. 2020, 10.1016/j.sjbs.2020.09.057. Accessed 10 Oct. 2021. 10.1016/j.sjbs.2020.09.057 PMC7783644 33424305

[29] Morren M et al. “Perceived Genetic Knowledge, Attitudes towards Genetic Testing, and the Relationship between These among Patients with a Chronic Disease.” *Patient Education and Counselling*, vol. 65, no. 2, Feb. 2007, pp. 197–204, 10.1016/j.pec.2006.07.005. Accessed 10 Nov. 2021. 10.1016/j.pec.2006.07.005 16939709

[30] Maftei A, Dănilă O. “The Good, the Bad, and the Utilitarian: Attitudes towards Genetic Testing and Implications for Disability.” *Current Psychology*, vol. 42, 17 Jan. 2022, 10.1007/s12144-021-02568-9 10.1007/s12144-021-02568-9 PMC8761521 35068904

[31] National Institute of Health(NIH)/National Center for Advancing Translational Sciences. “Hydrops Fetalis | Genetic and Rare Diseases Information Center (GARD) – an NCATS Program.” *Rarediseases.info.nih.gov*, rarediseases.info.nih.gov/diseases/2783/hydrops-fetalis. Accessed 10 Dec. 2022.

