# Supplementary material for: Awareness, attitudes towards genetic diseases and acceptability of genetic interventions among pregnant women in Burera district, Rwanda
Source: BMC Public Health. 2023 Oct 10;23:1961. doi: 10.1186/s12889-023-16866-3 (PMC10563347; doi:10.1186/s12889-023-16866-3)
Supplement: Supplementary file 1 — Supplementary Material 1 [file 12889_2023_16866_MOESM1_ESM.docx]

**Appendix. II QUESTIONNAIRE**

**II.1. DEMOGRAPHIC QUESTIONS**

| **SN** | **Question** | **Response** | **Code** |
| --- | --- | --- | --- |
| **1** | Age in years | Number: |  |
| **2** | Education | None  Primary  Secondary  Tertiary  Vocational | 1  2  3  4  5 |
| **3** | Marital status | Single  Married  Divorced | 1  2  3 |
| **4** | Religion | Christian  Muslim  Other | 1  2  3 |
| **5** | Have you been diagnosed with genetic disease | YES / NO  If yes, which one? …………………….. |  |
| **6** | Is there anyone in your family or the family of the baby's father who has been diagnosed with a genetic or inherited disorder? | YES / NO  If yes, which one? (optional)……………. |  |
| **7** | Do you live with any other chronic disease | YES / NO  If yes, which one? (optional)……………. |  |
| **8** | Have you ever had any birth defects | Yes / No  If yes, how many times. |  |
| **9** | Have you ever had Still birth | Yes / No  If yes, how many times |  |
| **10** | Do you smoke cigarette/tobacco? | YES / NO  If Yes, How often,:  -Daily  -Once a week  -Other, specify:……………… |  |
| **11** | Does your partner (husband)smoke? | YES / NO  If Yes, How often,  Daily  Once a week  Other, specify:……………… |  |
| **12** | Do you take alcohol? | YES / NO  If Yes, How often,  Daily  Once a week  Other, specify:……………… |  |
| **13** | Does your husband drink alcohol? | YES / NO  If Yes, How often:  -Daily  -Once a week  -Other, specify:……………… |  |
| **14** | Do you eat vegetables? | NO, Neither  Yes at Breakfast  Yes at Lunch  Yes at Dinner  At both meals  Other specify………  ………………………… | 1  2  3  4  5  6 |
| **15** | How often do you take fruits? | NO, Neither  Yes at Breakfast  Yes at Lunch  Yes at Dinner  At both meals  Other specify …………  ………………………… | 1  2  3  4  5  6 |
| **16** | Do you receive some iron from your Health facility when you are pregnant? | YES/ NO |  |
| **17** | Do you receive some Folic acid (Vit B9) from your Health facility when you are pregnant? | YES/ NO |  |
| **18** | Do you have a small home yard for vegetables at your home? | YES / NO |  |

**II. 2. OBSTETRIC QUESTIONS**

| **SN** | **Question** | **Response** | **Code** |
| --- | --- | --- | --- |
| **1** | Gestational trimester | First  Second  Third | 1  2  3 |
| **2** | Gravida, How many pregnancies have you ever had? | Number: |  |
| **3** | Parity (Total deliveries, including dead) | Number: |  |
| **4** | First visit to ANC | First  Second  Third | 1  2  3 |
| **5** | Have you ever had any birth defects | Yes / No  If yes, how many times. |  |
| **6** | Have you ever had Still birth | Yes / No  If yes, how many times |  |

**II.3. AWARENESS ABOUT GENETIC DISEASES, TESTING AND COUNSELLING**

**Select answers using Yes, No, Not sure**

| **No** | **Statements** | **Yes** | **No** | **Not sure** |
| --- | --- | --- | --- | --- |
|  | Have you ever heard about genetic diseases? | 1 | 2 | 3 |
|  | Do you know any inherited genetic diseases?  If yes, list any Two examples: ……………………………….. | 1 | 2 | 3 |
|  | Are genetic diseases contagious/infectious | 1 | 2 | 3 |
|  | Has any one of your family (3generations) suffered from a genetic disorder? | 1 | 2 | 3 |
|  | Which of the following increases the chance of developing genetic disease generations/offspring (from fetus, childhood to adulthood)? | | | |
|  | 1. Marriage between closed relatives | 1 | 2 | 3 |
|  | 1. Smoking for pregnant woman | 1 | 2 | 3 |
|  | 1. Exposure of secondary smoke to pregnant woman | 1 | 2 | 3 |
|  | 1. Taking alcohol during pregnancy | 1 | 2 | 3 |
|  | 1. Eating a balanced diet for a pregnant woman | 1 | 2 | 3 |
| Q6. | For a pregnant woman, which of the following can protect offspring from developing genetic disease? | | | |
|  | 1. Eating a balanced diet for a pregnant woman? | 1 | 2 | 3 |
|  | 1. Smoking | 1 | 2 | 3 |
|  | 1. Taking alcohol | 1 | 2 | 3 |
|  | 1. Taking iron (either in supplements or tablets) | 1 | 2 | 3 |
|  | 1. Taking Folic acid (Vitamin B9) | 1 | 2 | 3 |
| Q7. | Are you aware that there’s a specific test that confirms that the fetus is affected by a congenital malformation, Karyotyping, DNA typing using Invasive: Amniocentesis, cordocentesis, villus sampling? Non-invasive: Fetal plasma DNA) | 1 | 2 | 3 |

**II.4. ATTITUDES QUESTIONS ON GENETIC TESTING AND COUNSELLING**

**(NB: SA= Strongly agree, A= Agree; D= disagree; SD= strongly disagree; N= neutral)**

| ***SN*** | **Statements** | ***SA*** | ***A*** | ***N*** | ***D*** | ***SD*** |
| --- | --- | --- | --- | --- | --- | --- |
| ***Q1*** | Do you support pre-natal genetic testing as a way of screening for genetic diseases (When no typical problem is identified, like in **Ultrasound**)? |  |  |  |  |  |
| ***Q2*** | If, your child is identified to have a genetic disease, could you consent to induce abortion / pregnancy termination?  **……………………………………….** |  |  |  |  |  |
| ***Q3*** | Do you think it is right for parents to have genetic tests carried out for their children, for conditions that those children are at risk for, but as yet do not show symptoms for, and for which there is not currently a preventive treatment? |  |  |  |  |  |
| ***Q4*** | Do you think that it is necessary to go for genetic testing for a couple before getting married? |  |  |  |  |  |
| ***Q5*** | Do you think, the couple cannot proceed to get married if they found to have a genetic risk or a predisposition to genetic disease which can be passed on in their next generations? |  |  |  |  |  |
| ***Q6*** | In a given population, do you think it is a good idea to carry out carrier screening in order to identify people who are at risk of developing genetic disorders or passing them to their offspring? (Yes / No). |  |  |  |  |  |
| ***Q7*** | Do you support genetic counselling/do you think it is necessary? |  |  |  |  |  |
| ***Q8*** | Do you agree that some genetic diseases in family may cause persistent conflicts, family plans and goals not achieved, breaking marriage (like divorce etc…). |  |  |  |  |  |
| ***Q9*** | If during your pre-natal genetic testing, your child is screened/diagnosed with a serious fatal genetic disease, would you allow terminating pregnancy? |  |  |  |  |  |

**II.5. WILLINGNESS/ACCEPTABILITY QUESTIONS TOWARDS GENETIC TESTING AND COUNSELLING**

| **SN** | **Question** | **Response** | **Code** |
| --- | --- | --- | --- |
| **1** | Would you want to know if your baby could probably have a congenital/hereditary malformation? | Yes (1), No (2) |  |
| **2** | If performing this type of test (genetic testing) is available only in another hospital/clinic, will you agree moving to that hospital/clinic? | Yes (1), No (2) |  |
| **3** | If genetic screening is integrated as a standard procedure in Rwanda, will you support it? | Yes (1), No (2) |  |
| **4** | If a risk of a genetic disorder is identified in your pregnancy, or in you and your partner while screening, could you consent to do a Pre-implantation Genetic diagnosis for the next pregnancy? | Yes (1), No (2) |  |
| **5** | Could you consent for your child to be tested for Genetic disorders? | Yes (1), No (2) |  |
| **6** | Could you accept to discuss with your clinical geneticist/genetic counsellor about you and or members of the family about your risks (for example carrier risks, risks to current or future pregnancies or chance of being affected by the condition)? | Yes (2), No (2) |  |
| **7** | Have you ever sought genetic counselling? | Yes (2), No (2) |  |
| **8** | If you are identified to have a problem with conceiving naturally, would you support, would you allow using pre-implantation genetic diagnosis with In Vitro fertilization? (Egg and sperm are grown in vitro to produce a zygote and implanted again in the uterus) | Yes (2), No (2) |  |

Anything else you can share with the community regarding this study?............................................................................................................................................................................................................................................................................................................
